# Latent Memory of Unattended Stimuli Reactivated by Practice: An fMRI Study on the Role of Consciousness and Attention in Learning

**DOI:** 10.1371/journal.pone.0090098

**Published:** 2014-03-06

**Authors:** Julia D. I. Meuwese, H. Steven Scholte, Victor A. F. Lamme

**Affiliations:** 1 Brain and Cognition, Department of Psychology, University of Amsterdam, The Netherlands; 2 Amsterdam Brain and Cognition Centre (ABC), Amsterdam, The Netherlands; University of British Columbia, Canada

## Abstract

Although we can only report about what is in the focus of our attention, much more than that is actually processed. And even when attended, stimuli may not always be reportable, for instance when they are masked. A stimulus can thus be unreportable for different reasons: the absence of attention or the absence of a conscious percept. But to what extent does the brain learn from exposure to these unreportable stimuli? In this fMRI experiment subjects were exposed to textured figure-ground stimuli, of which reportability was manipulated either by masking (which only interferes with consciousness) or with an inattention paradigm (which only interferes with attention). One day later learning was assessed neurally and behaviorally. Positive neural learning effects were found for stimuli presented in the inattention paradigm; for attended yet masked stimuli negative adaptation effects were found. Interestingly, these inattentional learning effects only became apparent in a second session after a behavioral detection task had been administered during which performance feedback was provided. This suggests that the memory trace that is formed during inattention is latent until reactivated by behavioral practice. However, no behavioral learning effects were found, therefore we cannot conclude that perceptual learning has taken place for these unattended stimuli.

## Introduction

The brain is continually monitoring what happens in the world around us. Although we can only report about what is in the focus of our attention, much more than that is actually processed. Several studies show that without attention many early neural processes continue (e.g. [1], see for a review [2]), but this processing is too shallow to engage higher-order brain areas enabling a conscious report. For example, during ‘inattentional blindness’ (IB), when attention is directed towards a primary task, subjects fail to notice a secondary stimulus about which they were not informed [3], [4]. However, even though these unexpected stimuli are not reportable, perceptual processing of these stimuli is still intact in visual areas, such as contour integration or figure-ground segregation [5], [6]. Unattended stimuli are rich in content [7], [8] and have perceptual characteristics [9], [10]. Therefore, we think that unattended stimuli contribute to so-called ‘phenomenal consciousness’: the part of our conscious experience to which we do not have attentional access [11]–[14].

The other way around, even when attended, stimuli may not always be reportable. For instance, presenting a pattern mask shortly after a stimulus can render it fully invisible, even when it is attended ([15], [16], but note that object substitution masking is determined by attention to the mask instead of to the stimulus itself [17], [18]). Pattern backward masking blocks feedback processes, preventing the stimulus to reach a perceptual stage [19]. Yet, stimulus processing is not fully disrupted during masking. Even though masked stimuli are not consciously perceived, they can be processed up to our frontal brain areas in a feedforward manner, influencing our behavior (so called ‘masked priming’) ([20], [21], see for a review [22]).

This illustrates that, although attention and consciousness often go hand in hand, that does not mean that they are identical or indistinguishable processes. Recently, several other studies have shown that attention and consciousness can be dissociated theoretically and manipulated independently [11], [23]–[27]. There can be consciousness without attention (e.g. during IB), and attention without consciousness (e.g. during masking) [13]. In both conditions, despite a lack of reportability, stimuli are still being processed on a lower level, albeit in a different manner. During IB there is still perceptual processing in visual areas, during masking feedback processing is blocked.

The brain extracts information from the environment not only to act upon directly, but also to learn from experience. In order to make optimal decisions, it is key to adapt continually and become sensitive to important stimuli. In fact, our current percepts are shaped by our previous visual experiences [28]. Although it is obvious that the brain can learn from fully processed stimuli that are in the focus of attention [29]–[33], little is known about long-term consequences of different kinds of unreportable stimuli. For unattended, task-irrelevant stimuli perceptual learning has been shown [34], [35], but for attended yet masked stimuli, possible long-term effects of unconscious priming have never been investigated. We thus ask the following question: if a lack of reportability can be caused by either the absence of attention or the absence of consciousness, does this difference in stimulus processing have any differential long-term consequence?

Previously, we have tried to answer this question by independently manipulating consciousness and attention, measuring neural activity with electroencephalography (EEG) [36]. We exposed subjects to stimuli that were either masked yet attended, or unmasked yet not attended, which yields stimuli unreportable for orthogonal reasons. One day later, we assessed whether any neural and behavior learning had occurred for these stimuli. We found neural and behavioral learning effects only for the unattended stimuli. Remarkably, the behavioral learning effect became manifest after performance feedback was provided on the task measuring stimulus detectability. This suggests that the memory trace formed during inattention is latent until accessed.

Here, we further investigate the notion that exposure to unattended stimuli is sufficient to change the brain, whereas exposure to attended yet masked stimuli is not. We use functional magnetic resonance imaging (fMRI) to obtain insight in higher spatial detail compared to the EEG method used in our previous study. Also, we further explore – on a neural level – the observation that behavioral feedback can reactivate a latent learning trace. To this end, we added an extra neural measurement to the experimental design, one hour after the behavioral detection task with performance feedback was administered.

## Materials and Methods

### Participants

Twenty-nine subjects (all females) with no relevant psychiatric or neurological history participated in the experiment for course credit or financial compensation. We included only female participants, as it is our personal experience that they are generally more likely to follow instructions closely (especially important when rendering subjects ‘inattentionally blind’). Our results may therefore not generalize to male subjects, although so far the only known gender effects show more robust (task-irrelevant) perceptual learning effects in male compared to female subjects [37]. Subjects were all right-handed and had normal or corrected-to-normal vision. All subjects were screened on the possibility of metal in their bodies and other risk factors precluding participation in MRI studies. We obtained written informed consent from each participant before experimentation. The experiment was approved by the Ethical Committee of the Psychology Department of the University of Amsterdam.

Two subjects from the Inattention group were excluded because they reported to have seen the figure presented on day 1 (as assessed by the 10AFC task, see below). Two subjects from the Masked group were excluded: one because of chance performance on the day 1 ‘catch’ detection task, one because of abnormal brain anatomy. After exclusion, thirteen participants were tested in the Inattention group (*M* = 20.15 years of age, SD = 1.64 years), and twelve in the Masked group (*M* = 20.40 years of age, SD = 0.85 years).

### Task design

#### General procedure

**Figure 1:**
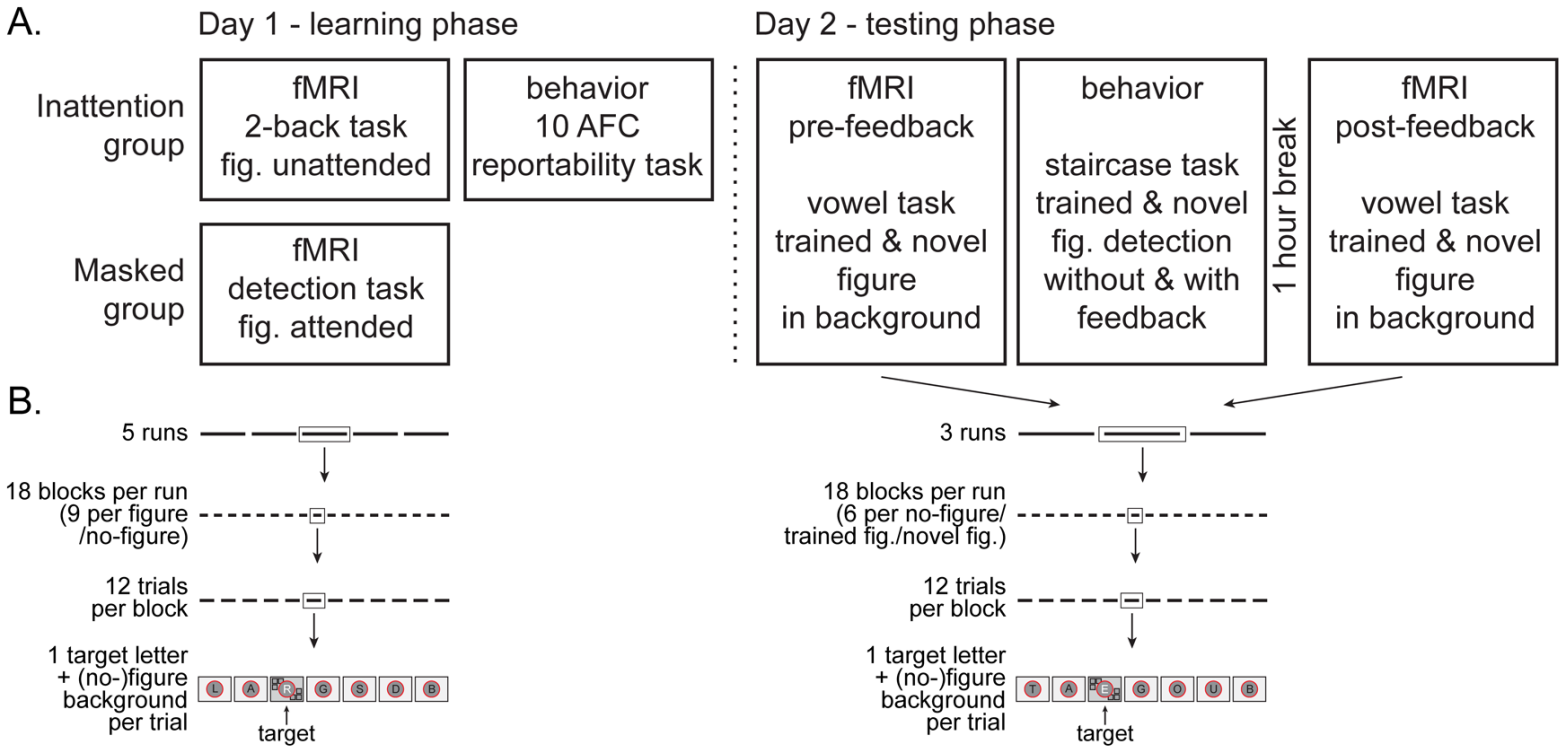
Schematic general procedure. **1A:** The experiment consists of two separate sessions on consecutive days; a learning phase (on day 1) and a testing phase (on day 2). During the learning phase, both groups are exposed to a letter stream, against a background of textured figures. These figures are unreportable in both groups, but for different reasons. The Inattention group performs an irrelevant letter task and hence ignores the textured background, resulting in so-called ‘inattentional blindness’ for the figures (checked with a surprise 10AFC task). The Masked group is trying to detect figures in the background, yet these figures are masked (except for luminance defined ‘catch’ figures), rendering them invisible even with attention. One day later, during the testing phase, both groups perform the same tasks. First, learning is assessed neurally by an easy letter task, while in the background both trained figures and novel figures are presented (measuring BOLD activity). Thereafter, a staircased detection task with trained and novel figures is administered without and with feedback, to measure (the effect of performance feedback on) behavioral learning effects. After a 1-hour break, post-feedback BOLD activity is measured using the same set-up as the first (pre-feedback) BOLD measurement, to assess neural effects of performance feedback. **1B:** Schematic illustration of the run, block and trial structure of each fMRI task. Trial details (exact timing and target stimulus specifications) can be found in Figure 2. Note that each block consists of a continuous letter stream, there are no breaks in between trials (only in between blocks and runs).

The experiment consisted of two separate sessions on consecutive days; a learning phase (on day 1) and a testing phase (on day 2) (see Figure 1A for the schematic procedure). Experimental setup was largely similar to Meuwese et al. [36], except for a few adjustments to make the experiment fMRI-compatible (instead of EEG) and additional neural measurements (second (‘post-feedback’) neural measurement on day 2 to examine the neural effect of behavioral feedback, plus a retinotopic mapping session one week later).

On day 1, every subject was assigned to either the Inattention or the Masked group on the basis of a predetermined randomization schedule. BOLD activity was recorded while both groups were exposed to the group specific stimuli. For both groups, stimuli consisted of a rapid serial visual presentation (RSVP) letter stream, presented against a background of textured figures. In both groups, the objective was that subjects were not able to report the presence of the textured figures, but for different reasons. To achieve that, the Inattention group had to perform a task related to the letter stream and hence ignore the textured background. This – together with not informing subjects in advance about the presence of the textured figures – results in the subjects not noticing the textured figures (see below for further details of this inattentional blindness manipulation). The Masked group could ignore the letter stream yet had to focus their attention on the textured background. To make stimuli escape reportability in this case, the figures of the textured background were masked (stimuli were not masked for the Inattention group, yet presented equally briefly). To engage attention on the background stimulus train, catch target stimuli were inserted between the masked texture figures, and subjects had to detect these. Note that our experimental design demanded the conditions to be assigned in a between-subject rather than within-subject manner; otherwise subjects who would be first assigned to the Masked condition would not be naïve to the presence of the figures in the Inattention condition, which would detrimentally render the figures reportable in this condition. Also on day 1, subjects of both groups were assigned to either subgroup A or subgroup B, indicating the position at which textured figures were presented. Figures presented on day 1 are called ‘trained’ figures.

On day 2, both groups performed the same easy (RSVP) letter task, while in the background both trained figures and novel figures (i.e. at quadrants not exposed before) were presented to assess learning neurally (measuring BOLD activity). Thereafter, a staircased detection task with trained and novel figures was administered to measure behavioral learning effects. To investigate the effect of performance feedback, behavioral learning without and with feedback was administered. After a 1-hour break, post-feedback BOLD activity was measured using the same set-up as the first (pre-feedback) BOLD measurement on the same day, to assess neural effects of performance feedback.

During a third session (on average 7 days later) a retinotopic localizer of our figure stimuli was presented, in addition to standard retinotopic mapping tasks, to determine ROIs.

#### Stimuli

**Figure 2:**
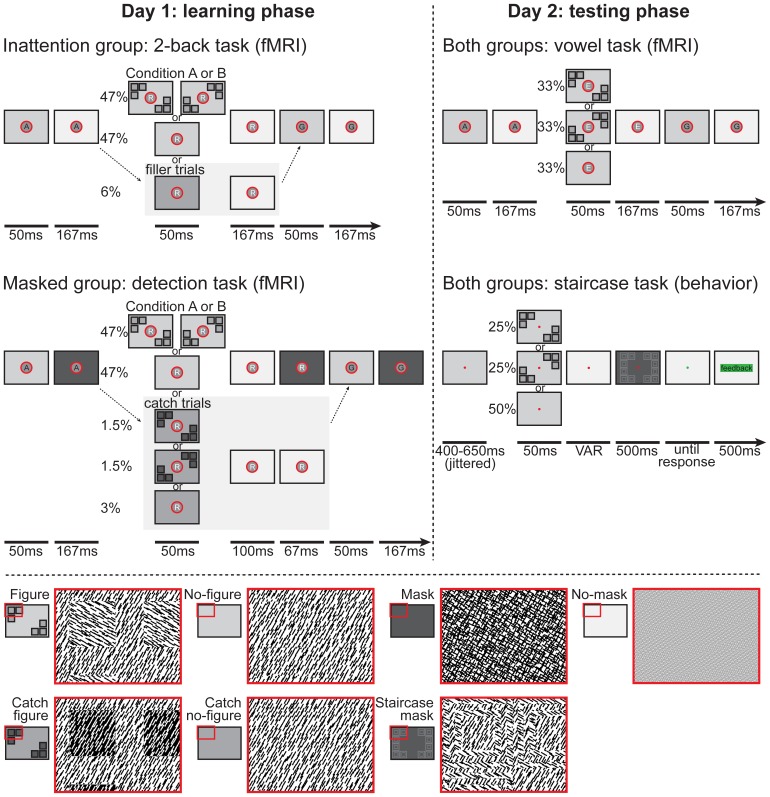
Schematic representation of the task designs. On day 1 (learning phase) both groups perform a different task. The Inattention group performs a RSVP 2-back letter task, while a textured pattern was presented that either formed a figure (containing 6 squares, in layout A or B) or a no-figure (containing no squares) is presented in the background together with every target letter. The Masked group performs a figure detection task, where they have to detect whether or not a masked figure texture is present in the background while the target letter is presented. Easily detectable unmasked luminance defined ‘catch’ figure trials (both layout A and B) have been added to keep subjects motivated, and to check whether the task instructions were being followed (in 6% of the trials, matched by 6% ‘dummy trials’ in the Inattention group). For both groups, figure and no-figure trials were presented in a block design of 12 consecutive figure or no-figure trials (every ‘trial’ being a sequence of 6–8 letters, with 1 white target letter which was paired with a figure/no-figure). On day 2 (testing phase) both groups perform the same two tasks, to measure learning effects neurally (vowel task) and behaviorally (staircase task). In the vowel task, subjects have to indicate whether the target letter is a vowel or not, while both the trained and novel figure (layout A and B) are presented in the background (again in a block design, similar to day 1). In the staircase task, subjects have to detect whether or not the masked target is a figure. Both the trained and novel figure and no-figures are now presented randomly. According to their performance the SOA between target and mask varies, in a double staircase manner for both figures independently. Two versions of the staircase task are presented, one without and one with performance feedback. Percentages refer to the percentage of all trials that a particular stimulus/trial type was presented. This figure has been adapted from our previous study, Meuwese et al. [36].

Stimuli were textured patterns identical to the ones used in Meuwese et al. [36]. Full field textured patterns were composed of patches of homogenously oriented line elements, oriented at either 30°, 60°, 120° or 150°. Target textures formed either a figure (containing 6 squares) or a no-figure (containing no squares). Texture defined figures contained a background with line elements oriented at either 30°, 60°, 120° or 150°, and 6 squares (individual width 3.6°) consisting of line elements oriented orthogonal to the background, divided over 2 diagonally opposite quadrants (3 squares in each quadrant, in 2 possible configurations (A or B), see Figure 2). In the two remaining quadrants another 6 squares were present, however their line elements had the same orientation as the background texture, so that they were not visible (although because of a different jittering of the line elements, very vague ‘boundaries’ between square and background are discernible on close scrutiny (see ‘no-figure’ panel in Figure 2)). The no-figure texture also contained 12 squares (3 in each quadrant), however all squares had the same orientation as the background texture and were hence invisible (except that again, different jittering of the line elements created vague ‘boundaries’ between square and background). The reason for doing this is to make figure and no-figure stimuli fully comparable from the point of view of low level features, such as line orientation and length (see also [38]). This enabled us to isolate neural activity specifically related to figure-ground segregation (and not just boundary detection) by subtracting no-figure from figure BOLD signals (for a similar procedure, see [6], [16], [39], [40]). Stimuli were created using Matlab (The MathWorks, Natick, MA, USA). They were back-projected on a 61×36 cm LCD screen using Presentation (Neurobehavioral Systems, Albany, CA, USA) and viewed through a mirror attached to the head coil.

### Day 1 – Learning phase

#### Greyscales task

Within each group (Inattention and Masked), participants were equally assigned to one of two conditions: A and B, referring to the position of the figures presented on day 1 (see Figure 2). Visual field biases (an asymmetry in representation and perception of/attention for one side of the visual field) may influence learning effects [41]. Therefore, before being assigned to a condition every subject was tested for visual field bias by performing a computerized version of the Greyscales task [42] (both the original version and a 90° rotated one, so biases could be calculated for both left versus right and top versus bottom of the visual field). In this task, subjects had to indicate which of two vertically aligned bars, the upper or the lower one, both shaded from dark to light but in a mirror reversed manner, appeared darker overall. The luminance difference between the two bars became increasingly smaller until, at the critical part of the task, for 72 trials overall luminance of the two bars was equal, unbeknownst to the subjects. An overall bias score could then be calculated from these trials (incorporating the horizontal and vertical bias, see ‘Behavioral data analysis’ section for details). Based on these overall bias scores, subjects were assigned to a condition such that visual field bias differences between conditions were minimized. The bias scores indeed did not differ between conditions (Inattention group: *t*_(1,11)_ = −.282, *p* = .78; Masked group: *t*_(1,10)_ = −.577, *p* = .58) and between groups (*t*_(1,23)_ = 1.438, *p* = .16).

#### Inattention group (2-back task)

We used an inattention paradigm largely similar to Meuwese et al. [36], based on the paradigm used by Scholte et al. [6]. It consisted of a primary stream of stimuli of which the subjects were informed (letters) and a secondary stream of stimuli of which the subjects were not informed (textured background patterns, forming either a figure or no-figure).

In more detail, the primary stream consisted of a foveally presented rapid serial visual presentation (RSVP) of letters at a speed of 217 ms per letter (A, G, P, S, R, O, L, B, D and T were used), which were presented on a red fixation point with a grey center of 1.1° in diameter. These letters were usually black, but once every 6–8 letters, a white target letter was presented. Subjects were instructed to indicate whether every white letter was the same as or different from the white letter presented two instances earlier (2-back task), by pressing one of two buttons of a response box (using index and middle finger of the right hand, counterbalanced across subjects). At the onset of every white letter a short beep sounded, to minimize the number of missed responses.

The secondary stream of stimuli consisted of textured patterns that were presented with the onset of each new letter for 50 ms (the remaining 167 ms an isoluminant grey dotted pattern was presented). Together with every black letter a homogenous pattern was presented. However, at the onset of each white letter a textured pattern was presented that either formed a figure or a no-figure (see “Stimuli” section above).

Figure and no-figure trials were presented in a blocked design, throughout 5 runs. There were a total of 18 blocks per run, 9 blocks containing figure trials and 9 blocks of no-figure trials. Each block consisted of 12 trials (every ‘trial’ being a sequence of 6–8 letters, of which 1 white target letter was presented together with a figure/no-figure) (see Figure 1B). The order of figure and no-figure blocks was counterbalanced across runs (and the order of runs was counterbalanced across subjects). In 12 out of the 18 blocks per run, one ‘filler’ trial was presented, which was a homogenous textured pattern to match the ‘catch’ trials in the Masked group paradigm (see below for further explanation of ‘catch’ trials). In total 1080 trials were presented (510 per figure/no-figure, 60 filler trials), with a total duration of approximately 40 minutes (including breaks).

Each run started with 16 seconds fixation, after which four 8-letter dummy trials were presented (7 seconds total). In these dummy trials, an RSVP letter stream was presented without any target letters or background textures, in order to keep fixation and baseline attention level constant. In between each block there was a short ‘rest period’ during which two dummy trials were presented (3.5 seconds total). Each run ended with four dummy trials and a 16 seconds fixation period. Subsequent background textures always contained line elements with a different orientation to ensure that a new image was presented with the onset of each new letter (black or white). The number of figures and no-figures was equal for all background orientations, and each orientation was equally divided over the white letters and figure and no-figure trials. The task was preceded by a short practice version (12 trials, with only no-figure backgrounds) outside the scanner to make subjects acquainted with the task.

We evaluated the presence or absence of inattentional blindness for the textured figures at the end of the session, by means of a surprise 10 alternative forced choice (10AFC) task. Subjects were told that one figure texture had been presented regularly during the 2-back task, at the onset of the white letter, and they were instructed to select it from a set of 9 other figure textures (see Figure S1). The actual figures (both A and B configurations) and 8 others consisting of circles and rectangles with similarly oriented line elements and textures of 0° and 90° were presented in random order (numbered 1 to 10). They were asked to choose one of these options, even if they had to guess. If subjects failed to select the correct figure, they were considered to be unable to report about the figure, i.e. to have suffered from inattentional blindness during the exposure to the texture figures. Two out of 15 subjects did choose the correct figure (although they reported they were simply guessing), and they were excluded from further analysis.

#### Masked group (Detection task)

Subjects in the Masked group were presented with a paradigm similar to the Inattention group, however there were some crucial differences. Our aim was to manipulate reportability of the figure by masking instead of inattention. Therefore, all background textures were masked (the mask consisting of two superimposed homogenous textures with orthogonally oriented line elements) (see ‘mask’ panel in Figure 2). Masking was achieved by having each figure/no-figure stimulus (50 ms), being followed by an isoluminant grey screen (for 100 ms), followed by a mask with a duration of 67 ms.

Instead of the 2-back letter task, subjects directed their attention towards the secondary stream of stimuli to perform a figure detection task. With every white letter (accompanied by a short beep), they had to indicate whether or not a figure was present in the background, by pressing one of two buttons of a response box (using index and middle finger of the right hand). Because of the mask, this task was practically undoable (but that was indeed our goal, unreportability of the figures by masking, even with attention). To keep our subjects motivated, and to check whether the task instructions were being followed, “catch trials” were presented in 6% of the trials, which were much easier to detect. These “catch trials” contained unmasked and fairly visible luminance defined ‘catch’ figures (both layout A and B, to prevent any learning effects of location to occur, and to ensure fixation at the center of the screen) and ‘catch’ no-figures. These were presented in 12 out of the 18 blocks per run (equally distributed over figure and no-figure blocks), to keep the ratio of ‘catch’ trials equal to the set-up of our previous (EEG) study [36] and to prevent anticipation effects that would arise if a ‘catch’ trial occurred in every block. Note that in the Inattention group, to keep number of trials and task length equal, ‘filler trials’ were presented instead of these ‘catch trials’, containing only ‘catch’ no-figures (see Figure 2).

### Day 2 – Testing Phase

On day 2, subjects of both groups (Inattention and Masked) performed the same tasks, to measure learning effects neurally (Vowel task) and behaviorally (Staircase task). A novel figure was now introduced (its spatial configuration opposite to the trained figure, presented on day 1) to compare its neural signature and behavioral detectability with that of the trained figure.

In contrast to day 1, for both groups the figure stimuli were now reportable (for the Masked group because they were no longer masked, for the Inattention group because they had been told at the end of day 1 that there had been a figure present in the background). To prevent subjects from directing their attention towards the now visible background figures during the Vowel task (see below), we briefly showed them both the trained and novel figure backgrounds at the start of day 2 (for 1 second each, in random order, without telling them which one was presented on day 1). They received the explicit instruction that they should not pay any attention to these figures, even though they would appear in the background during the Vowel task.

#### Pre-feedback BOLD measurement (Vowel task)

Subjects of both groups (Inattention and Masked) performed the same task, to measure neural BOLD activity evoked by both the trained figure and a novel figure. Subjects performed an RSVP letter task to ensure fixation in the center, while being passively exposed to figures and no-figures in the background. The task was similar to the task performed by the Inattention Group on day 1, except that here for every white letter (A, E, O, U, S, T, G and B were used), subjects had to indicate whether it was a vowel or a consonant, by pressing one of two buttons of a response box (using index and middle finger of the right hand, counterbalanced across subjects). The task is easy, and different from the 2-back task (Inattention group, day 1) to ensure equal task difficulty for both groups. With every white letter (as on day 1, accompanied by a short beep), either a figure (A or B) or no-figure was presented, which allowed us to make relevant subtractions in the BOLD signal in order to assess neural learning effects.

In total 648 trials were presented (216 per figure A/figure B/no-figure) in 3 runs containing 18 blocks each (6 blocks per figure A/figure B/no-figure), with a total duration of approximately 24 minutes (including breaks). The rest of the set-up is identical to day 1 (see above), except that there was no need for any filler/catch trials now (as both groups now performed the same letter task).

#### Behavioral detection task (Staircase task)

All subjects performed the same task, to measure behavioral detectability of the trained and the novel figure. Every trial a target, either a figure (A or B) or no-figure, was presented (50 ms, accompanied by a short beep), followed by an isoluminant screen (variable duration, starting at 350 ms) and a meta-contrast mask consisting of orthogonally oriented line elements (500 ms, see ‘staircase mask’ panel in Figure 2). This meta-contrast mask was chosen instead of the pattern mask used during the learning phase (in the Masked condition) as it is a stronger mask that can block stimulus processing over a wider range of SOA's more effectively, resulting in a more sensitive measure of stimulus detectability (this mask could not be used in the learning phase however, as it contains square shapes that would confound possible learning effects). After presentation of the mask the red fixation dot turned green to indicate that a response could be made. Subjects then pressed one of two buttons of a response box (using index and middle finger of the right hand, counterbalanced across subjects), to indicate whether or not they saw a figure preceding the mask. When no response was given 1500 ms after mask presentation, a warning message appeared ‘No response! Respond when the fixation dot is green’ (but in Dutch) which stayed on screen until a response was made.

The stimulus onset asynchrony (SOA) between the target and the mask was initially set at 350 ms for both figures, but varied in a double staircased manner (for each figure independently), according to the subject's responses. For every two correct responses the SOA was shortened by 17 ms, for every incorrect response it was lengthened by 17 ms, thereby varying the duration of the isoluminant screen in between target and mask. The SOA of the no-figures was paired with both figures (trained and novel, in a 50/50 manner), such that with every SOA there was an equal probability for a figure or no-figure as a target. After 60 trials for each figure/no-figure pair, this resulted in two separate SOA curves, one for the trained figure and one for the novel figure.

Subjects performed this task twice in a row. The first time as described above, the second time performance feedback was given at every trial: after each response, a green or red bar appeared (for 500 ms), with the words ‘correct’ or ‘incorrect’ respectively.

In total 120 trials were presented in 1 block, with a duration of approximately 6 minutes. The task was performed inside the scanner (although no BOLD activity was measured) to keep all conditions equal to the learning phase. Before subjects were placed inside the scanner they performed a short practice version of the behavioral detection task (10 trials, with only no-figure backgrounds) to make subjects acquainted with the task.

#### Post-feedback BOLD measurement (Vowel task)

One hour after subjects completed the Staircase task with performance feedback, they were placed back into the scanner to perform the Vowel task (see above, “Pre-feedback BOLD measurement (Vowel task)”) again. The exact same set-up was used, in order to compare the pre- and post-feedback BOLD signal for the trained versus the novel figure. Run order was randomized and counterbalanced across subjects.

### Behavioral Data analysis

Behavioral data were analyzed using SPSS 17.0 (Statistical Product and Service Solutions, IBM, Armonk, USA). For day 1, bias scores on the Greyscales task were calculated as in Nicholls et al. [42], by subtracting leftward responses from rightward responses, divided by the total number of responses (idem for bottom minus top responses for the 90° rotated version). The horizontal and vertical bias scores (both ranging from −1 to +1) were then multiplied, resulting in one overall bias score. Overall bias score differences between conditions and groups were tested with independent t-tests. Performance on the 2-back task (Inattention group, day 1) was calculated in percentage correct. For the detection task (Masked group, day 1) performance (percentage correct and d-prime) was analyzed separately for the catch stimuli and the masked stimuli. It was tested with paired two-tailed t-tests whether these performances differed significantly from chance.

Equal performance on the Vowel task (day 2) was tested with an independent t-test between both groups (percentage correct). Behavioral learning effects on day 2 were established by comparing the results of the Staircase task for the trained versus the novel figure, for both the feedback and no feedback version. For each group, the trials were binned to 5 bins (12 trials each), and for every 5^th^ bin (the end point of the SOA curves) a paired t-test was performed for the trained versus the novel figure.

### fMRI acquisition and analysis

#### fMRI Acquisition

We used a 3T Philips Achieva TX MRI scanner at the Spinoza Center in Amsterdam. A high-resolution 3DT1-weighted anatomical image (TR, 8.175 ms; TE, 3.74 ms; FOV, 240×220×188, 1 mm^3^ voxel size, 2 averages) was recorded for each subject at the beginning of the first session (day 1). On day 2 and during the third session (retinotopic mapping) the same 3DT1 scan was made for coregistration purposes, but now only with one repetition. BOLD-MRI was recorded on day 1 and day 2 using gradient-echo, echo-planar imaging (TR 2000 ms, TE 27.63 ms, FA 76.1, 37 slices with ascending acquisition, voxel size 3 mm^3^, slice gap 0.3 mm, FOV 240×240×122). During retinotopic mapping, functional MRI was recorded using a gradient-echo, echo-planar pulse sequence (TR 2000 ms, TE 34.54 ms, FA 76.1, 24 slices with ascending acquisition, voxel size 2.5 mm^3^, slice gap 0.25 mm, FOV 66×200×144) centered around the calcarine sulcus. An MRI-compatible eyetracker (EyeLink 1000, SR Research, Canada) was used to monitor whether subjects maintained fixation throughout the tasks.

#### Region of Interest localization

Visual areas were localized using a polar mapper and an eccentricity mapper. The polar mapper consisted of a checkerboard wedge (red-green tiles, flickering at 6 Hz) rotating around fixation (2 counterclockwise and 2 clockwise runs) and the eccentricity mapper was a checkerboard ring (red-green tiles, flickering at 6 Hz) expanding from center to periphery (plus one run contracting from periphery to center). During these runs, subjects fixated at the center while detecting blue tiles flickering amidst the red-green checkerboard tiles (pressing buttons for either left/right side of the screen (polar) or close/far (eccentricity)). The TR of these two runs was set to 2500 ms as the phase of the wedge and expanding/contracting ring was set at 2500 ms (6 phases resulted in one cycle of 15 seconds).

In addition to the retinotopic mappers, a stimulus-specific localizer was used. This localizer consisted of a checkerboard pattern (four red-green tiles, flickering at 7.5 Hz) forming 6 squares identical in layout and size to our textured figure stimuli, on a black background. Each figure was presented for 8 seconds, and was repeated 16 times throughout one run. Throughout the task subjects fixated at the center and detected blue tiles flickering amidst the red-green checkerboard tiles (pressing buttons for left/right side of the screen).

We used these retinotopic and stimulus-specific localizers such that for each subject, data of these runs were projected onto an inflated surface reconstruction of the subject's brain (using Brainvoyager 2.1 (Brain Innovation, Maastricht, The Netherlands [43])). Retinotopic mappers allowed us to define visual areas (V1, V2, V3 and V4), whereas the stimulus-specific localizer was used to determine voxels responding to either the trained or novel figure locations (configuration A and B, counterbalanced across subjects, see Figure 2). First, figure-specific locations (responsive to either configuration A or B) were defined for each visual area separately by selecting overlapping voxels, resulting in trained figure and novel figure-specific subregions for each visual area. Then, final ROIs were determined by the 10% highest t-values of the figure-specific localizer within each figure-specific subregion. This resulted in a series of ROIs that, depending on whether subjects were assigned to subgroup A or B, were equivalent to locations where the figure was presented on day 1 or not (trained figure V1–V4, novel figure V1–V4).

In the Masked group, the stimulus-specific localizers did not evoke figure specific activity in V4 for four subjects (trained figure for one subject, novel figure for three other subjects), such that no figure-specific subregion of V4 could be defined for these figures. In these cases, the V4 ROI was defined by the 10% highest t-values of the figure-specific localizer in whole V4, instead of only the figure-specific subregion of V4.

The number of voxels did not differ between the trained figure and novel figure ROI (Inattention group: *t*_(1,12)_ = 1.610, *p* = .13; Masked group: *t*_(1,11)_ = .701, *p* = .50) or between groups (trained figure ROI: *t*_(1,23)_ = −.273, *p* = .79; novel figure ROI: *t*_(1,23)_ = −.320, *p* = .75).

For the analysis of the fMRI data of the learning phase (day 1), for each of the ROIs a GLM were fitted to determine figure and no-figure related activity. Obviously, on day 1, a difference between figure and no-figure activity is only to be expected at ROI's where figures are shown (A or B, depending on the subject). For the analysis of fMRI data from the testing phase (day 2), we also fitted a GLM on each ROI to determine trained figure, novel figure and no-figure BOLD activity for each visual area (V1–V4), now averaging over A and B locations.

#### BOLD signal analysis

Data were analyzed using Brainvoyager 2.1 (Brain Innovation, Maastricht, The Netherlands [43]) and Matlab (The MathWorks, Natick, MA, USA). Functional scans were slice-time corrected, motion corrected, spatially smoothed with a Gaussian of 2 mm FWHM (full width at half maximum) and high-pass filtered using a 0.01 Hz FFT (Fast Fourier Transform) filter. All functional scans were aligned to the first functional scan, which was coregistered to the T1-weighted anatomical image. Functional scans from day 2 and the retinotopic mapping session were also coregistered to the T1 image of the first session (day 1) (after first coregistering to the T1 image of its own session, as T1 images are easier to coregister than functional scans). Structural images were transformed to Talairach space using an ACPC (Anterior Commissure Posterior Commissure) transformation [44].

Functional data from day 1 (learning phase) were analyzed as follows: for each of the ROIs (A and B locations, V1–V4) a General Linear Model (GLM) with 2 predictors (figure and no-figure) was fitted to determine figure and no-figure related activity. The GLM was modeled in each subject and ROI separately for the five experimental runs combined (for one subject in the Inattention group the GLM was modeled over three runs instead of five, as only three runs were recorded due to technical difficulties). To investigate within-group differences in figure versus no-figure processing, an ANOVA was run on the beta-values for each group separately (factors: stimulus type (figure, no-figure) and ROI (V1, V2, V3, V4)). To assess between-group differences, a similar ANOVA was used (stimulus type×ROI), with between-subject factor group (Inattention, Masked). All these ANOVAs were performed for the beta-values of the appropriate A or B locations, as explained above.

Functional data from day 2 (testing phase) were analyzed in a similar fashion. First figure processing was investigated for the pre-feedback and post-feedback runs separately (fitting a GLM with trained figure, novel figure and no-figure as predictors for each ROI). To assess within-group differences, three ANOVAs (factors: stimulus type×ROI) were performed to compare processing of the trained and novel figure versus no-figure and versus each other (one ANOVA for trained figure versus no-figure, one for novel figure versus no-figure, one for trained figure versus novel figure), for pre-feedback and post-feedback runs separately. The same ANOVAs were run to investigate between-group differences in trained and novel figure processing, this time with group (Inattention, Masked) as a between-subject factor, also for both pre-feedback and post-feedback runs.

To compare the pre-feedback BOLD measurement with the post-feedback BOLD measurement, three ANOVAs were performed (factors: feedback (pre-feedback BOLD signal, post-feedback BOLD signal) and ROI (V1, V2, V3, V4)), separately for the trained figure – no-figure BOLD signal, novel figure – no-figure BOLD signal and trained figure – novel figure BOLD signal. The same ANOVAs were used to assess between-group differences, adding group (Inattention, Masked) as a between-subjects factor.

## Results

### Learning Phase (Day 1)

#### Behavioral results: manipulation checks

**Figure 3:**
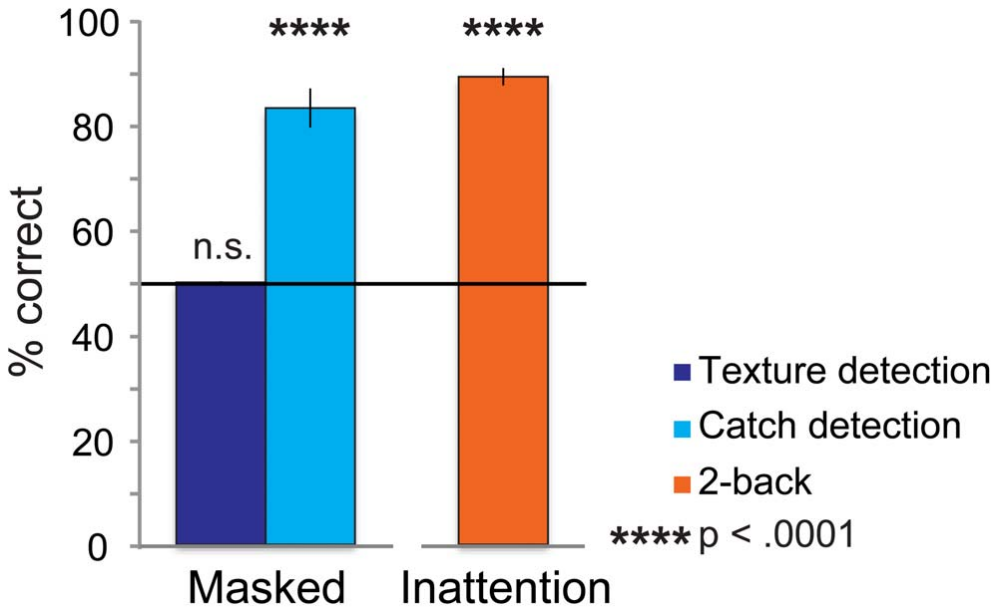
Behavioral performance on day 1 (learning phase). For the Masked group, percentage correct does not differ significantly from chance for detection of masked figure textures (49.9% correct, *t*_(1,11)_ = −.423, *p* = .68), whereas unmasked ‘catch’ figures are detected above chance level (83.2% correct, *t*_(1,11)_ = 8.901, *p*<.0001), which indicates that subjects were performing the task correctly but just were not able to detect the masked figure textures. The Inattention group performs above chance level at the 2-back letter task (89.2% correct, *t*_(1,12)_ = 23.65, *p*<.0001), indicating that top-down attention was directed at the letter task instead of the background figures.

For the Inattention group performance on the 2-back task was significantly better than chance (89.2%, *t*_(1,12)_ = 23.65, *p*<.0001) (see Figure 3). Attention was thus successfully directed towards the primary stream of stimuli, which rendered the figure presented in the secondary stimulus stream unreportable for most subjects (except the two out of 15 subjects who chose the correct figure in the 10AFC task and were excluded from further analysis (see also ‘Participants’ of the Methods section)).

Subjects in the Masked group were indeed attending to the background to perform the figure detection task, as detection of the catch trials was significantly better than chance (83.2%, *t*_(1,11)_ = 8.901, *p*<.0001). The actual figures were successfully masked, as detection of these masked figures was at chance level (49.9%, *t*_(1,11)_ = −.423, *p* = .68) (see Figure 3). The d-prime (d′) value for detection of the masked figures (d′ = −0.08) also did not differ significantly from chance (i.e. a d′ value of 0) (*t*_(1,11)_ = −1.60, *p* = .14).

#### fMRI results: more figure related processing in the Inattention group

**Figure 4:**
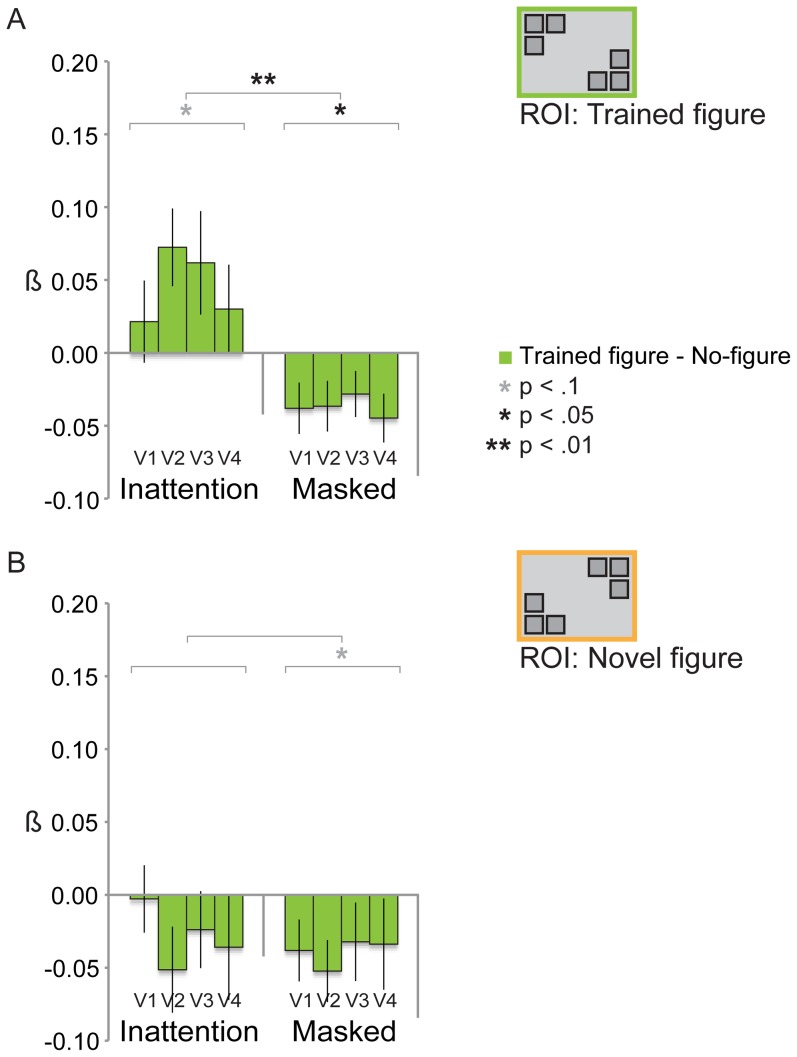
Trained figure-evoked BOLD signal on day 1 (learning phase). **5A:** In the ROI of the trained figure, in the Inattention group, a higher BOLD signal can be observed for the trained figure (minus no-figure) evoked signal than in the Masked group (F_(1,23)_ = 7.945, *p* = .0097). Within the Inattention group, the trained figure evoked signal differs from the no-figure signal at trend level (F_(1,12)_ = 3.361, *p* = .092). Thus, figure-ground segregation processes proceeded during inattention (see also Meuwese et al., 2013; Scholte et al., 2006), although marginally. For the Masked group, trained figure-related activity is blocked by the mask, such that the trained figure evoked even a lower BOLD signal than no-figure (F_(1,11)_ = 7.111, *p* = .02, note that this is an effect in the opposite direction; less figure activity than no-figure activity). The mask thus successfully blocked figure processing, which is also reflected in the performance on the detection task (which is at chance level, see Figure 3). **5B:** In the ROI of the novel figure (which is not yet presented on Day 1) as expected, the trained figure evoked BOLD activity does not differ from that of the no-figure, neither within (Inattention group: F_(1,12)_ = 1.175, p = .30; Masked group: F_(1,11)_ = 3.736, p = .08 (although note that this is an effect at trend level in the opposite direction: less trained figure than no-figure evoked activity)) nor between groups (F_(1,23)_ = .100, p = .75).

Figure textures were processed differently in both groups (see Figure 4). In the Inattention group, a higher BOLD signal can be observed for the figure minus no-figure evoked signal than in the Masked group (F_(1,23)_ = 7.945, *p* = .0097). Within the Inattention group, the figure evoked signal differs from the no-figure signal at trend level (F_(1,12)_ = 3.361, *p* = .092). Thus, figure-ground segregation processes proceeded during inattention (see also [36] and [6]), although marginally. For the Masked group, figure-related activity was blocked by the mask, such that figure evoked even a lower BOLD signal than no-figure (F_(1,11)_ = 7.111, *p* = .022, note that this is an effect in the opposite direction; less figure activity than no-figure activity). The mask thus successfully blocked figure processing, which was also reflected in the chance-level performance on the detection task (see above).

As expected, in the ROI where no figure was presented (ROI A or B, depending on the subject), figure evoked activity did not differ from that of the no-figure, neither within nor between groups (see Figure 4). This indicates that the figure versus no-figure signal described above is location specific, and not some sort of general arousal effect.

### Testing Phase (Day 2)

#### Behavioral results: manipulation checks

During the BOLD measurements of the testing phase, both groups performed the same task (Vowel task, see Figure 2). Performance was equal for both groups, for the pre-feedback Vowel task (Inattention group: 96.0%, Masked group: 95.0%; *t*_(1,23)_ = .795, *p* = .43) as well as the post-feedback Vowel task (Inattention group: 96.9%, Masked group: 94.2%; *t*_(1,23)_ = 1.794, *p* = .09, although this p-value is at trend level). Within groups, there was no difference between pre- and post-feedback Vowel task performance (Inattention group: *t*_(1,12)_ = −1.294, *p* = .22, Masked group: *t*_(1,11)_ = 1.309, *p* = .22). These findings imply that group differences in neural figure processing (see below) cannot be caused by differences in Vowel task performance, as performance was equal for both groups.

#### Behavioral results: no learning effect present in Staircase task

**Figure 5:**
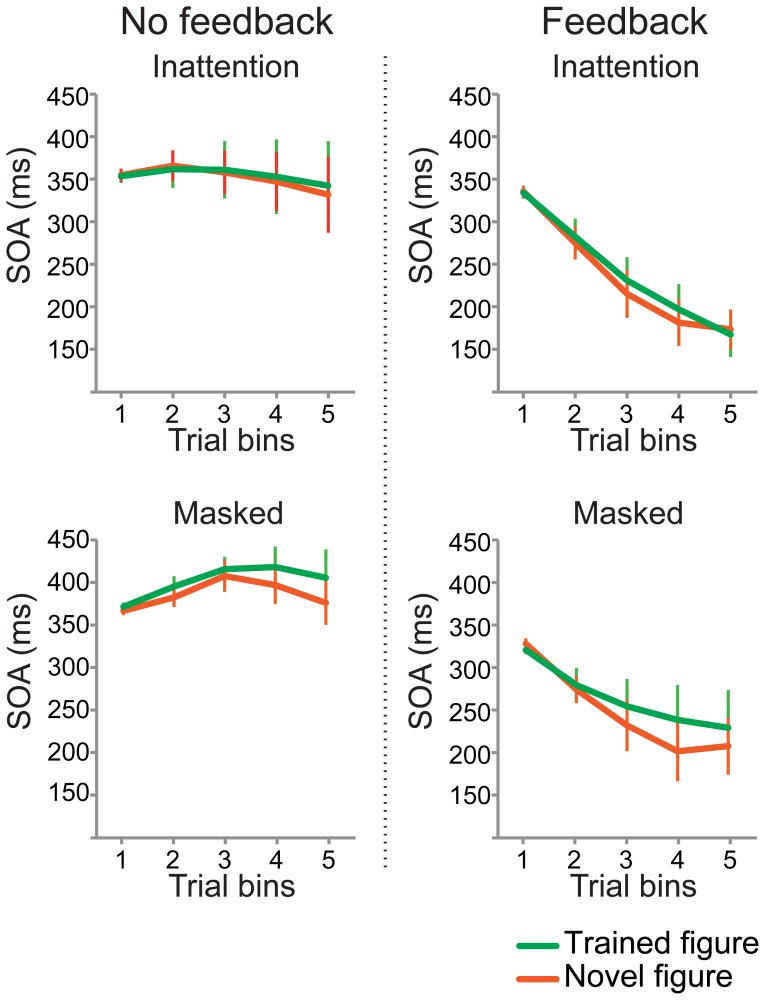
Behavioral performance on the Staircase task on day 2 (testing phase). No learning effects were found in the Staircase task, for either the version without or with performance feedback (Inattention group, no feedback: *t*_(1,12)_ = .462, *p* = .65, feedback: *t*_(1,12)_ = −.595, *p* = .56; Masked group, no feedback: *t*_(1,11)_ = 1.103, *p* = .29, feedback: *t*_(1,11)_ = .549, *p* = .59). There was no effect of feedback on the learning effect (trained minus novel figure)) (Inattention group: *t*_(1,12)_ = .709, *p* = .49, Masked group: *t*_(1,11)_ = .319, *p* = .76).

No learning effects (difference in SOA of 5^th^ bin between trained versus novel figures) were found in the staircased behavioral detection task, for either the version without or with performance feedback (Inattention group, no feedback: *t*_(1,12)_ = .462, *p* = .65, feedback: *t*_(1,12)_ = −.595, *p* = .56; Masked group, no feedback: *t*_(1,11)_ = 1.103, *p* = .29, feedback: *t*_(1,11)_ = .549, *p* = .59) (see Figure 5). Furthermore, there was no effect of feedback on the learning effect (trained minus novel figure) (Inattention group: *t*_(1,12)_ = .709, *p* = .49; Masked group: *t*_(1,11)_ = .319, *p* = .76).

Thus, contrary to our previous findings [36], we did not find a significant effect of feedback on detectability of the trained figure in the Inattention group. Moreover, we cannot conclude that any perceptual learning has taken place, as that is defined as an increased ability to extract sensory information from the environment [45].

#### fMRI results: increased BOLD signal only for trained figure in Inattention group for post-feedback measurement

**Figure 6:**
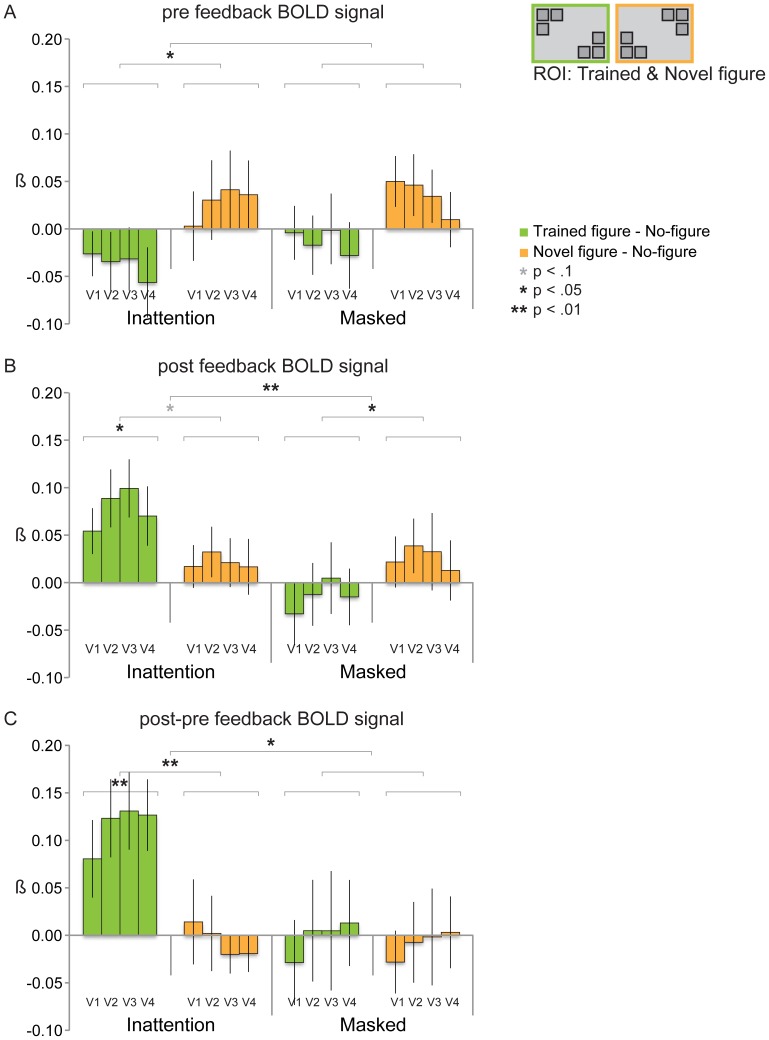
Trained figure and novel figure-evoked BOLD signal on day 2 (testing phase). **6A:** We did not find a significant neural learning effect in the Inattention group for the pre-feedback BOLD measurement. For both groups, figure activity did not differ significantly from no-figure activity, for both the trained figure (Inattention group: F_(1,12)_ = 1.595, *p* = .23; Masked group: F_(1,11)_ = .154, *p* = .70) and the novel figure (Inattention group: F_(1,12)_ = .569, *p* = .47; Masked group: F_(1,11)_ = 1.670, *p* = .22). Nor did we find any learning effects (trained figure versus novel figure) in the BOLD signal for both groups. In fact, there was more novel figure than trained figure activity in both groups, even significantly so in the Inattention group (F_(1,12)_ = 5.902, *p* = .03; Masked group: F_(1,11)_ = 2.590, *p* = .14). Between-groups, we did not find any significant differences. **6B:** The post-feedback BOLD signal for the trained figure was significantly higher than for the no-figure in the Inattention group (F_(1,12)_ = 8.512, *p* = .013), not in the Masked group (F_(1,11)_ = .188, *p* = .67) (between group difference: F_(1,23)_ = 4.929, *p* = .037). Trained figure activity was higher than novel figure activity in the Inattention group at trend level (F_(1,12)_ = 3.561, *p* = .08), in the Masked group the trained figure evoked a significantly lower signal than the novel figure (F_(1,11)_ = 8.136, *p* = .02) (between group difference: F_(1,23)_ = 8.110, *p* = .009). In both groups, novel figure activity does not deviate from no-figure activity. **6C:** When post- and pre-feedback BOLD signal are compared, we found a large post-feedback increase of BOLD activity for the trained figure and for the learning effect (trained figure minus novel figure), only in the Inattention group (trained figure: F_(1,12)_ = 9.807, *p* = .009, learning effect: F_(1,12)_ = 13.099, *p* = .004) (Masked group, trained figure: F_(1,11)_ = .001, *p* = .98, learning effect: F_(1,11)_ = .048, *p* = .83). Between groups, the BOLD signal increase of the trained figure differs at trend level (F_(1,23)_ = 3.620, *p* = .07), the increase of the learning effect (trained versus novel figure) differs significantly (F_(1,23)_ = 6.023, *p* = .02). For the novel figure there were no significant BOLD changes in the post-feedback signal compared to the pre-feedback signal in both groups.

Contrary to our expectations, we did not find a significant neural learning effect in the Inattention group for the first, pre-feedback, BOLD measurement (see Figure 6A). For both groups, figure activity did not differ significantly from no-figure activity, for the trained figure (Inattention group: F_(1,12)_ = 1.595, *p* = .23; Masked group: F_(1,11)_ = .154, *p* = .70) as well as the novel figure (Inattention group: F_(1,12)_ = .569, *p* = .47; Masked group: F_(1,11)_ = 1.670, *p* = .22). Nor did we find any learning effects (trained figure versus novel figure) in the BOLD signal for both groups. In fact, there was more novel figure than trained figure activity in both groups, even significantly so in the Inattention group (F_(1,12)_ = 5.902, *p* = .03; Masked group: F_(1,11)_ = 2.590, *p* = .14). Between-groups, we did not find any significant differences either (trained figure: F_(1,23)_ = .338, *p* = .57; novel figure: F_(1,23)_ = .025, *p* = .88; trained figure versus novel figure: F_(1,23)_ = .195, *p* = .66).

Enhanced BOLD activity for the novel compared to the trained figure may suggest that in the Inattention group some sort of adaptation effect (e.g. a long-term version of the well known repetition priming [46], [47]) has occurred for the trained figure, and/or that a novelty effect was present for the novel figure. Although for the Masked group the difference between trained and novel figure BOLD signal is far from significant, relatively enhanced activity for the novel figure may indicate a small novelty effect in this group as well. However, in both groups none of the figure-related activity deviates significantly from no-figure activity, so speculations about possible novelty and adaptation effects should be regarded with caution.

Whereas the first (pre-feedback) BOLD measurement (and the behavioral Staircase task) did not show any learning effects, the second (post-feedback) BOLD measurement (taking place one hour after the Staircase task) showed increased activity only for the trained figure, and only for the Inattention group (see Figure 6B). It is striking how in the Inattention group the trained figure-specific signal has evolved from a negative BOLD signal during the pre-feedback measurement, to a significantly positive signal during the post-feedback measurement (F_(1,12)_ = 8.512, *p* = .013), whereas novel figure evoked activity again did not differ from no-figure activity (F_(1,12)_ = .857, *p* = .37). In the Masked group however, both the trained and novel figure still evoked a signal that did not differ from the no-figure signal (trained figure: F_(1,11)_ = .188, *p* = .67; novel figure: F_(1,11)_ = .782, *p* = .40). On the between-group level, trained figure activity was higher in the Inattention group than in the Masked group (F_(1,23)_ = 4.929, *p* = .037), whereas no group difference was observed for the novel figure (F_(1,23)_ = .016, *p* = .90). Thus, a significant figure-ground signal was found only for the trained figure, only in the Inattention group, and this effect differs significantly from the Masked group.

When comparing the post-feedback BOLD signal of the two figures, trained figure activity was higher than novel figure activity in the Inattention group at trend level (F_(1,12)_ = 3.561, *p* = .08). But remarkably, in the Masked group the trained figure evoked a significantly *lower* signal than the novel figure (F_(1,11)_ = 8.136, *p* = .02) (although both figure signals did not deviate from the no-figure signal in the Masked group, see above). Thus, there is a positive difference between trained and novel figure signal in the Inattention group (although p-value is at trend level), whereas this difference is negative in the Masked group. These opposing effects differ significantly on the between-groups level (F_(1,23)_ = 8.110, *p* = .009).

When post- and pre-feedback BOLD signals were compared (see Figure 6C), we found a large post-feedback increase of BOLD activity for the trained figure and for the learning effect (trained figure minus novel figure), only in the Inattention group (trained figure: F_(1,12)_ = 9.807, *p* = .009, learning effect: F_(1,12)_ = 13.099, *p* = .004). No difference between post- and pre-feedback BOLD signal was observed in the Masked group at all (trained figure: F_(1,11)_ = .001, *p* = .98, learning effect: F_(1,11)_ = .048, *p* = .83). Between groups, the BOLD signal increase of the trained figure differs at trend level (F_(1,23)_ = 3.620, *p* = .07), the increase of the learning effect (trained versus novel figure) differs significantly (F_(1,23)_ = 6.023, *p* = .02). For the novel figure there were no significant BOLD changes in the post-feedback signal compared to the pre-feedback signal in both groups (Inattention group: F_(1,12)_ = .020, *p* = .89; Masked group: F_(1,11)_ = .049, *p* = .83; between group difference: F_(1,23)_ = .002, *p* = .96).

In sum, neural learning is absent in the pre-feedback signals, apart from a novelty effect in the Inattention group (more signal for the novel figure than the trained figure, although activity for both figures does not deviate from no-figure activity). In the post-feedback signals however, we observe three different learning effects. Firstly, there is a significant figure-ground signal only for the trained figure in the Inattention group, which differs significantly from the trained minus no-figure signal of the Masked group. Secondly, there is a marginally significant difference (p-value at trend level) between trained figure and novel figure activity in the Inattention group, and this learning effect differs significantly from the Masked group. Finally, in the Masked group, there is an opposite effect: trained figure signal is *lower* than that of the novel figure (significantly so in the post-feedback BOLD signal), which could be some sort of adaptation effect due to long-term repetition priming (although both figures do not deviate from no-figure signal). When pre- and post-feedback signals are compared, no difference at all is observed for the Masked group. For the Inattention group, the difference between pre- and post-feedback learning is highly significant, at multiple levels (for trained minus no-figure signal alone, but also for trained versus novel figure activity and the difference of this learning effect compared to the Masked group). The strongest effect we observe, therefore, is an effect of the behavioral detection task on learning contingent on the subjects being exposed to figures during Inattention. We interpret this as exposure to figures during Inattention building a latent memory trace in visual cortex that only becomes neurally manifest after subjects practice figure detection and receive feedback on their performance.

## Discussion

We found increased figure-ground BOLD activity to figure stimuli one day after exposure to figure stimuli at the same location, but only when this prior exposure occurred during inattention, and not for prior exposure to masked figure stimuli to which attention was directed. Remarkably, this BOLD increase only became apparent after a behavioral detection task was administered to assess learning, during which performance feedback was provided, even though behaviorally no learning effect was apparent. These results suggest that the memory trace formed during inattention is latent until accessed, and reactivation of this trace by behavioral practice (a detection task with performance feedback) is needed for neural consolidation.

### Effect of behavioral practice with performance feedback

Interestingly, we only found a neural learning effect for the Inattention group after a behavioral detection task had been administered during which performance feedback was provided. It seems that this practice stage is needed to make a latent neural learning trace (formed during inattention) measurable at the level of BOLD MRI. It is hard to draw conclusions about the exact mechanisms underlying this BOLD increase, as we did not include a condition in which subjects did not receive feedback on the second run of the Staircase task (or for that matter, a condition in which the entire behavioral practice was absent). Thus, we do not know for sure whether the observed neural effects are a *direct* result of the performance feedback or just a result of practice (by (repeating) the behavioral task). Yet, we would like to speculate that that the measured BOLD augmentation is boosted by the performance feedback, as in our previous EEG study [36], performance feedback brought forward a behavioral learning effect (which was the reason for adding the post-feedback BOLD measurement to the current study). In any case, we deem it unlikely that mere passive exposure to the figure stimuli would be sufficient to cause the post-feedback BOLD increase, as one would then expect to see a gradual BOLD increase throughout the exposure, in both pre- and post-feedback fMRI sessions, instead of the sudden increase we observe now. Even if mere passive exposure/active practice without feedback would be sufficient to cause neural augmentation of a learning trace, we think that performance feedback will serve to boost this effect, just as it can boost the speed and extent of learning effects behaviorally [48], [49].

Although, as said above, in the current experiment we cannot prove the direct causal relation between performance feedback and bringing forward the neural learning effect, we would like to speculate about the underlying mechanisms if indeed feedback plays a role in boosting neural learning. How did performance feedback possibly activate this latent memory trace, one day after it was formed? We think that feedback may play an important role in consolidating reactivated memory traces. Dobres and Watanabe [50] were the first to show that feedback and consolidation, two mechanisms involved in learning that are usually studied independently, actually interact powerfully. They conclude that in fact, consolidation of learning is a *fundamental effect* of feedback. Many studies (including the current study) assess learning by retesting the trained stimulus together with a novel stimulus. Retesting reactivates the neural memory trace of the trained stimulus, rendering it in a fragile state, requiring subsequent reconsolidation [51]. When reactivated, even consolidated traces are vulnerable to interference effects of the novel stimulus, which may in fact deteriorate learning effects. This interference with reactivated memory traces can even be so strong as to block previously learned information altogether, for instance by updating fear memories with non-fearful information during the reconsolidation window [52]. The reconsolidation window not only offers an opportunity to rewrite or erase memories, it can be used to strengthen learning traces as well. Dobres et al. [50] showed that whenever feedback was provided during training, this caused stabilization of learning, making the learning trace resilient to interference and deterioration during testing. This consolidating effect of feedback was specific for the trained stimulus and did not generalize across similar stimuli (dots moving in an orthogonal direction), and it was irrespective of the amount of learning. Even though Dobres et al. provided feedback during training over the course of several days (instead of only during testing, like we did in the current study (on day 2)), their findings support our idea that feedback is important for boosting learning effects, when reactivating previously formed memory traces. We think that performance feedback can strengthen the reconsolidation of reactivated memory traces, even if this feedback is provided one day after the initial learning trace has formed.

### Neural versus behavioral learning effects

In perceptual learning, behavioral and neural effects only become measurable after substantial training (namely, thousands of trials on many consecutive days, and usually with full attention) [33], [35], [53]–[55], but not necessarily simultaneously. Supposedly, the neural changes related to perceptual learning are present, and accumulating during the course of the thousands of trials. Our learning phase consists of far fewer trials than what is common in the perceptual learning literature, because usage of the inattentional blindness paradigm limited us to only one short learning phase (note that in similar task-irrelevant learning paradigms (e.g. [35]) unreportability of the to-be-learned stimulus was not crucial to the experimental design, allowing for a much longer training period). It is therefore not necessarily surprising that we do not find any behavioral effects, while we do find neural. Perhaps a much longer training period would induce behavioral learning, even in the Masked group [56], as learning of masked stimuli may simply be slower than learning of unattended stimuli. On the other hand, as we only find neural differences between groups for the post-feedback fMRI measurement and not for the pre-feedback fMRI session, the change in neural activity for the Inattention group could even be a merely temporary effect. Further research, including a longer training period and/or a post-training measurement at a later time point, is necessary to examine the solidity of the found neural changes, and presumably the occurrence of behavioral learning effects at a later stage.

### Task-irrelevant versus task-relevant learning

The so-called ‘task-irrelevant perceptual learning’ paradigm (TIPL, [34], [35], [57]) shows that learning can occur for task-irrelevant stimuli. The TIPL paradigm is quite similar to our Inattention condition, where figure stimuli are task-irrelevant as well, except that with TIPL stimuli are merely unattended, but not necessarily unreportable. It is known that task-irrelevant learning occurs only when the task-irrelevant stimulus is weak [58], [59]. Whenever stimulus strength is too strong, its processing is thought to be suppressed to prevent distraction, whereas a stimulus can escape this suppression when it is too weak to be ‘noticed’ by the attentional system at all. Presumably, in our study learning effects would have been stronger if our figure stimuli would have been weaker. However, it is crucial to our experimental setup that our stimuli are unreportable only because of a lack of attention. Instead of weak or even subliminal stimuli, we therefore chose stimuli strong enough to be surely reportable *with* attention.

Nonetheless, the TIPL paradigm may provide an alternative explanation for our findings. According to studies employing this paradigm, learning is thought to occur only when the task-irrelevant stimulus is temporally paired with a task-relevant stimulus, which triggers an internal reward [34], [60], [61]. This internal reward is a release of neuromodulatory factors that boost sensory signals, resulting in reinforcement learning. In our study, internal rewards might have been present in the Inattention group (triggered by the ‘task relevant’ letters, temporally paired with the ‘task irrelevant’ background figures), whereas in the Masked group such internal rewards paired to the target stimuli might have been less prominent, as task performance for masked figure detection was at chance level. This may explain our finding of learning during inattention and not masking. On the other hand, Seitz et al. [49] show that internal reinforcement signals are not necessarily related to correct performance. Furthermore, it has been shown that learning occurs when a target and stimulus are temporally paired (irrespective of task-relevancy of the stimulus), such that task-driven and stimulus-driven signals coincide [60], [62]. This temporal pairing is equal for both groups in our experiment.

In the Masked group, for the post-feedback BOLD measurement, trained figure signal was *lower* than that of the novel figure. Although activity for both figures did not deviate from no-figure activity, this could indicate that some sort of adaptation effect has occurred for the trained figure. It could be a long-term version of repetition priming [46], [47], [63]. Alternatively, this possible *inhibition* of learning may have been caused by the fact that in the Masked group attention was directed towards the figure stimuli during the learning phase (whereas in the Inattention group it was not). Namely, it has been shown that attention can interrupt learning [34], [64]. However, this only applies to *task-irrelevant* stimuli, whose processing is suppressed when distracting attention away from the *task-relevant* stimuli [58], [59], [65], [66]. Our figure stimuli are *task-relevant* in the Masked group, and task-relevant learning with attention is a well-established effect [67]–[71] (but see [72]–[74] for some rare exceptions with complex stimuli). Nonetheless, it must be noted that figure stimuli were *task-irrelevant* in the Inattention group, and this asymmetry in terms of task-relevancy may (partly) account for the opposite effects found in both groups.

### Invisible yet attended

How do we know whether the masked stimuli were really attended by the Masked group, during the learning phase? In object substitution masking, for example, it has been shown that successful masking is determined by attention to the mask instead of attention to the stimulus [17], [18]. These stimuli were presented in a visual search paradigm, however, where attention typically quickly hops from one location to the next. Here, we used a different type of masking (pattern masking), and included ‘catch’ stimuli at the same location and with a spatial layout and timing identical to the masked stimuli. These ‘catch’ stimuli served to motivate subjects to continuously attend to the location of the background stimuli. ‘Catch’ detection performance was well above chance, which indicates that the locations of the masked stimuli were indeed attended. Does this guarantee attentive processing of these invisible stimuli? Naccache et al. [75] showed that when masked stimuli are presented at the same location as visible stimuli, subsequent processing is affected by those invisible primes. Importantly, this does not happen for invisible primes presented at an unattended location, indicating that attention to visible stimuli at a particular location transfers to other –invisible- stimuli at that same location.

### fMRI versus EEG observations

The current study is a follow-up to our previous EEG study, in which we found neural and behavioral learning effects for unattended stimuli but not for masked yet attended stimuli, using a largely similar paradigm [36]. Although comparing EEG with fMRI results is fraught with problems, particularly in terms of temporal and spatial resolution, we attempt to reconcile our findings here. The EEG measurement on day 2 revealed a difference in neural processing between the trained and the novel figure for the Inattention group, which we interpreted as a neural learning effect. However, in the pre-feedback BOLD measurement we observed a novelty effect for this group (more BOLD activity for the novel compared to the trained figure). As the polarity of an EEG effect is not informative of the direction of the effect, this EEG effect may actually be interpreted as a novelty effect as well. It could also be the case that the EEG and BOLD results reveal different aspects of the same process. Possibly, the high temporal resolution of the EEG measurements picks up on fast, learning related differences (related to faster access to the trained stimulus for which a memory trace is present), whereas the BOLD measurements reveal ‘slow’ novelty effects (arising because of longer, divergent processing required for the novel stimulus). Alternatively, but less likely, we observe opposite effects in both studies (that may be due to different ROI selections/the different spatial scale of both techniques). Furthermore, in the EEG study we found a behavioral learning effect after feedback was provided on the detection task, whereas in the current study we do not measure any perceptual learning. This could be due to the fact that subjects were exposed to very few trials (see ‘Neural versus behavioral learning effects’ section above), even fewer trials compared to our EEG study (for fMRI-compatibility reasons we used a blocked design instead of randomized trials with 510 trials per figure instead of 680). Despite this lack of a perceptual learning effect, one hour later we do find a large increase of BOLD activity for the trained figure in the Inattention group. Unfortunately we do not know what would have happened to the EEG effect after the feedback brought forward the behavioral learning effect, as there was no post-feedback EEG measurement in our previous study. Thus, using fMRI and including a second neural measurement on day 2 has provided insight into the neural effect of reactivation of a memory trace (even though this trace is so fragile that it did not become apparent behaviorally), and the direction of the neural learning effects.

### Conclusions

We found differential neural learning effects for unreportable unattended stimuli and masked yet attended stimuli. Positive learning effects were observed in the Inattention group, whereas negative adaptation effects were found in the Masked group. This shows that different kinds of unreportability (perceptual or attentional blindness) can lead to different neural outcomes. The present findings suggest that behavioral practice (and/or, as we propose, performance feedback) plays a role in bringing forward the neural learning effect, as we found a large increase of the neural learning effect in the post-feedback BOLD signal, only for the Inattention group, even though behaviorally no learning or feedback effect was found. Additional study is required to further investigate the duration of this effect, the role of feedback, and whether results are valid to other types of tasks and stimuli as well. How many unreportable things do we learn, that are latent until accessed?

## Supporting Information

Figure S1 **Figures presented during the 10 AFC task to check for inattentional blindness.** This set of figure textures was presented (in random order, numbered 1 to 10) during the 10AFC task, in which subjects from the Inattention group had to select which figure had been presented in the background during the 2-back task they just finished. If subjects failed to select the correct figure, they were considered to have suffered from inattentional blindness for the figure presented in the background on day 1. Two subjects who succeeded to select the correct figure were excluded from further analysis, even though they reported they were merely guessing. Figure A and B are depicted in the bottom two pictures. This figure has been adapted from our previous article, Meuwese et al. [36], where we used an identical 10AFC task.
(TIF)
