# Supplementary figures and images for: Latent Memory of Unattended Stimuli Reactivated by Practice: An fMRI Study on the Role of Consciousness and Attention in Learning
Source: PLoS One. 2014 Mar 6;9(3):e90098. doi: 10.1371/journal.pone.0090098 (PMC3946088; doi:10.1371/journal.pone.0090098)

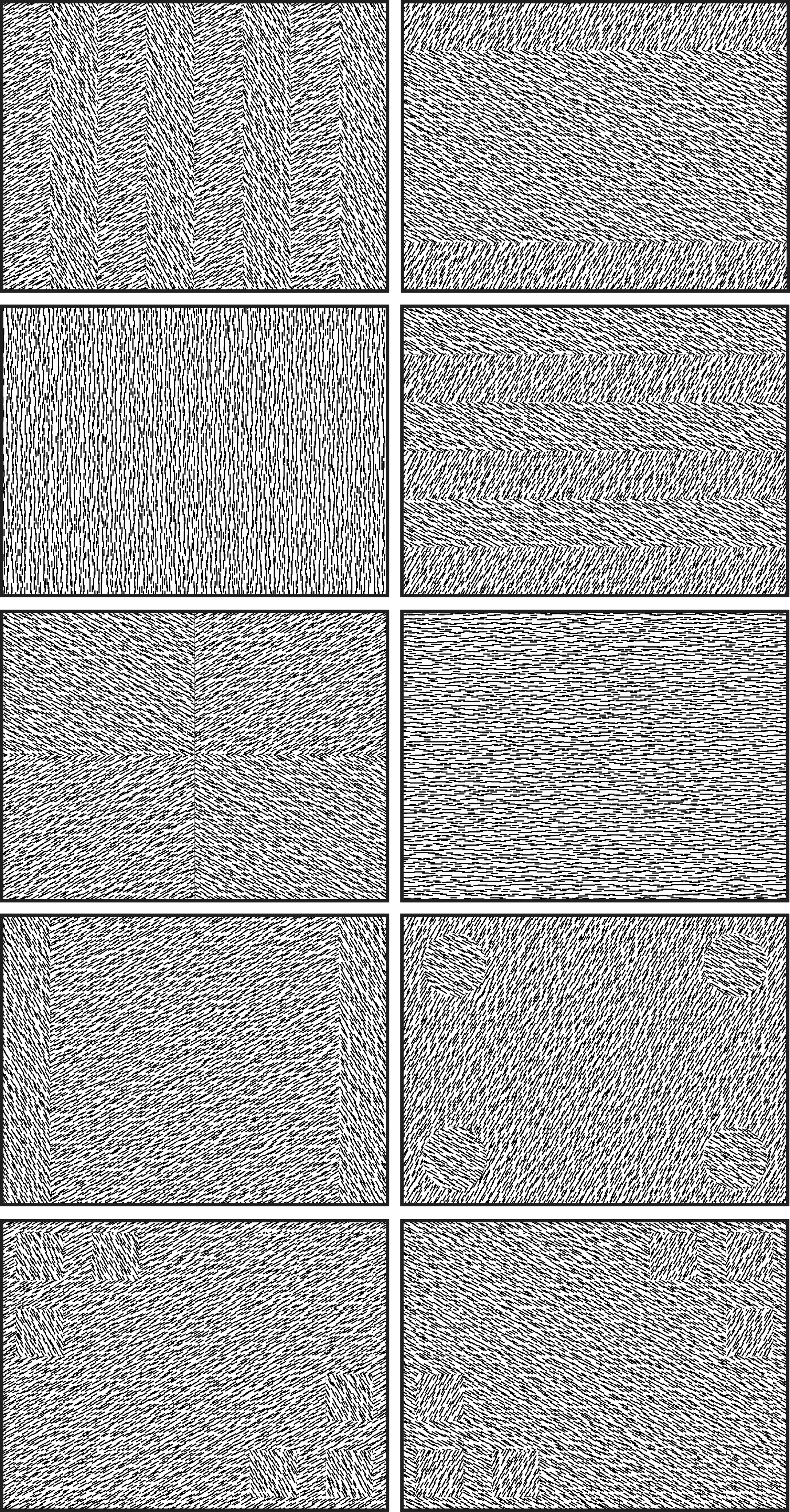

Supplement: Figure S1 — Figures presented during the 10 AFC task to check for inattentional blindness. This set of figure textures was presented (in random order, numbered 1 to 10) during the 10AFC task, in which subjects from the Inattention group had to select which figure had been presented in the background during the 2-back task they just finished. If subjects failed to select the correct figure, they were considered to have suffered from inattentional blindness for the figure presented in the background on day 1. Two subjects who succeeded to select the correct figure were excluded from further analysis, even though they reported they were merely guessing. Figure A and B are depicted in the bottom two pictures. This figure has been adapted from our previous article, Meuwese et al. [36], where we used an identical 10AFC task. (TIF) [file pone.0090098.s001.tif]
